# Hereditary multiple exostoses with a giant osteochondroma degenerated into chondrosarcoma

**DOI:** 10.1016/j.radcr.2024.04.012

**Published:** 2024-05-03

**Authors:** Federica Masino, Manuela Montatore, Rossella Carpentiere, Marina Balbino, Rossella Gifuni, Giacomo Fascia, Giuseppe Guglielmi

**Affiliations:** a Department of Clinical and Experimental Medicine, Foggia University School of Medicine, Viale L. Pinto 1, 71121, Foggia , Italy; b Radiology Unit, “Dimiccoli” Hospital, Viale Ippocrate 15, 70051, Barletta, Italy; c Radiology Unit, “IRCCS Casa Sollievo della Sofferenza” Hospital, Viale Cappuccini 1, 71013 San Giovanni Rotondo, Italy

**Keywords:** Hereditary multiple exostoses, Chondrosarcoma, Exostosis, Osteochondromas, Bone disorders, Diagnostic imaging

## Abstract

We present a case of hereditary multiple exostoses with malignant transformation to chondrosarcoma in a woman complaining of enlargement and pain in the right thigh. Hereditary multiple exostoses is a rare genetic disorder characterized by multiple osteochondromas. Malignant transformation to chondrosarcoma of a pre-existing osteochondroma is a possible significant manifestation of this hereditary syndrome. Imaging modalities such as X-ray, Ultrasound, and computed tomography play a crucial role in the diagnosis and management of these patients, as described in this case.

## Introduction

Hereditary multiple exostoses (HME), also known as multiple osteochondromas, is a rare autosomal dominant skeletal disorder with almost complete penetrance (95%). The prevalence is estimated at 1 in 50,000 people. This condition is characterized by the formation of multiple osteochondromas, also known as exostoses, which are benign bony outgrowths [1]. The WHO's fifth edition classification of soft tissue and bone tumors defined the criteria to diagnose the pathology that includes: 1) at least 2 osteochondromas at the juxta-epiphyseal area of the long bones radiologically diagnosed; 2) a positive family history and/or a germline mutation in the EXT (exostosin glycosyltransferase) genes which encode enzymes involved in the synthesis of heparan sulphate proteoglycans [2]. In particular, the chromosomes involved by genetic mutations in HME are 8q24 (EXT1), 11p11-13 (EXT2) and 19p (EXT3) [3].

Apart from hereditary reasons, osteochondroma development can also be attributed to trauma, surgery, and radiation exposure. When the epiphyseal plate is damaged, undifferentiated cartilage migrates into the metaphysis, leading to the development of exostosis, which mimics congenital osteochondromas both pathologically and radiographically. Though its exact cause is unknown, ectopic cartilage growth plate development is believed to cause osteochondroma production: after the separation from the growth plate, the epiphyseal cartilage herniates into the periosteal bone cuff [4].

The diagnosis of HME involves a multidisciplinary approach. Initial diagnosis of HME can be made performing imaging methods, with plain radiographs representing the gold standard to detect bone abnormalities and exostoses. However, magnetic resonance imaging is necessary for higher resolution, particularly in cases when surgical exostosis excision is planned. Genetic testing that pinpoints the disease-causing mutation provides definitive confirmation. Fundamental is the pedigree of the patient's family members and the whole exome sequencing for genetic mutations of EXT1, EXT2, and EXT3 [5,6].

Except for the cranial theca, all bones in the body can be affected by HME. In particular, osteochondromas most frequently originate from the appendicular skeleton, in half cases at the lower limb and around the knee. The most common locations are the distal femur (30%) and proximal tibia (15%-20%) followed by wrists and hands, humerus, ankle, pelvis, and ribs [1,7].

Most osteochondromas remain asymptomatic, but they can sometimes cause pain, deformity, and functional limitations, particularly when they impinge on surrounding structures. In case of complications, cross-sectional imaging is required. Malignant transformation of exostoses, although rare, is a well-recognized complication of HME, with chondrosarcoma arising from the cartilage cap of a previous osteochondroma being the most common and most feared consequence of HME. Imaging modalities such CT and MRI plays a crucial role in the evaluation of complications related to HME, with MRI being the gold standard for the characterization of malignant lesions transformation [5,8]. The fundamental radiological aspect to be evaluated in the suspicion of a malignant degeneration of osteochondroma in chondrosarcoma is the cartilage cap and its thickness. This is a parameter that can be assessed with greater accuracy with MRI [5].

## Case presentation

A 66-year-old woman with a known history of HME came to our clinic. The patient presented a familiar history of HME without complications. She complained of pain in the right thigh that started about 20 days earlier and had been progressively worsening. She reported surgical treatment for leg lengthening. She denied any recent trauma or injury to the area.

**Fig. 1:**
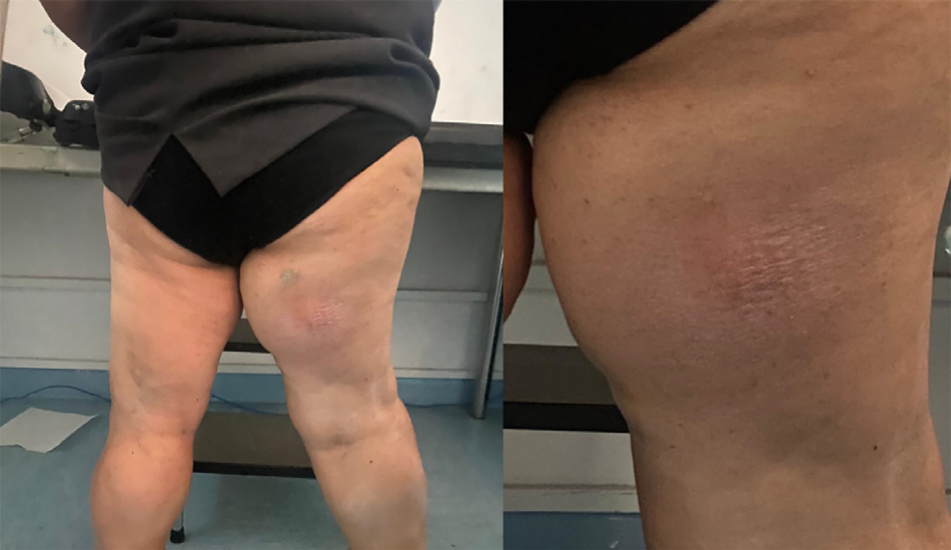
A posterior view of legs. It showed the different volumes with an enlargement of the right thigh.

On clinical examination, the patient presented difficulty walking with a limp due to dysmetria of the lower limbs (right longer than left) and joint deformities. The right thigh presented a noticeable enlargement compared to the contralateral side because of a voluminous mass extending anteroposteriorly from the inner root until the third middle. On palpation, both a softer and a harder component were perceived, with the first detected in the lower portion, where the patient reported pain on acupressure, and the second in the posterosuperior portion (Fig. 1).

**Fig. 2:**
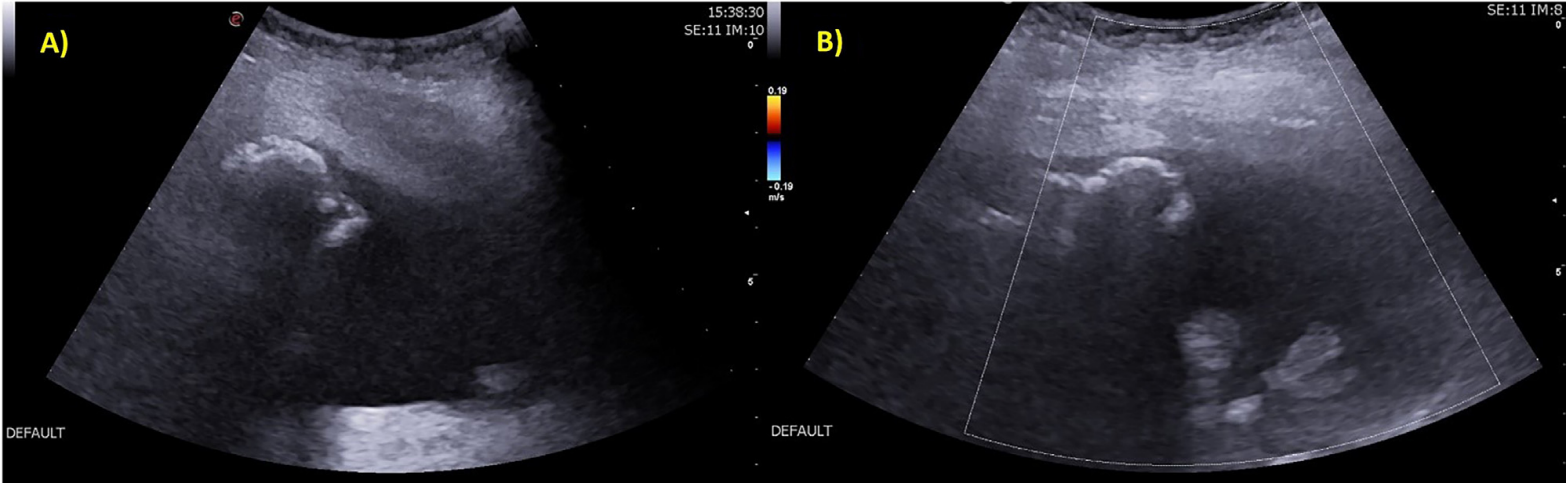
(A, B) US performed in orthostasis with the probe positioned at the posterior portion of the thigh, in the painful area reported by the patient. US in B-mode (A) showed a heterogeneous lesion with different types of content, with margins that were not definable. At Doppler application (B) there was no intralesional vascular signal.

Ultrasound (US) examination was performed in orthostasis and revealed the presence of a heterogeneous mass with multiple calcified and fluid components not recognizable as intra or extra-lesional. Doppler application did not show intralesional increased vascularity (Fig. 2).

**Fig. 3:**
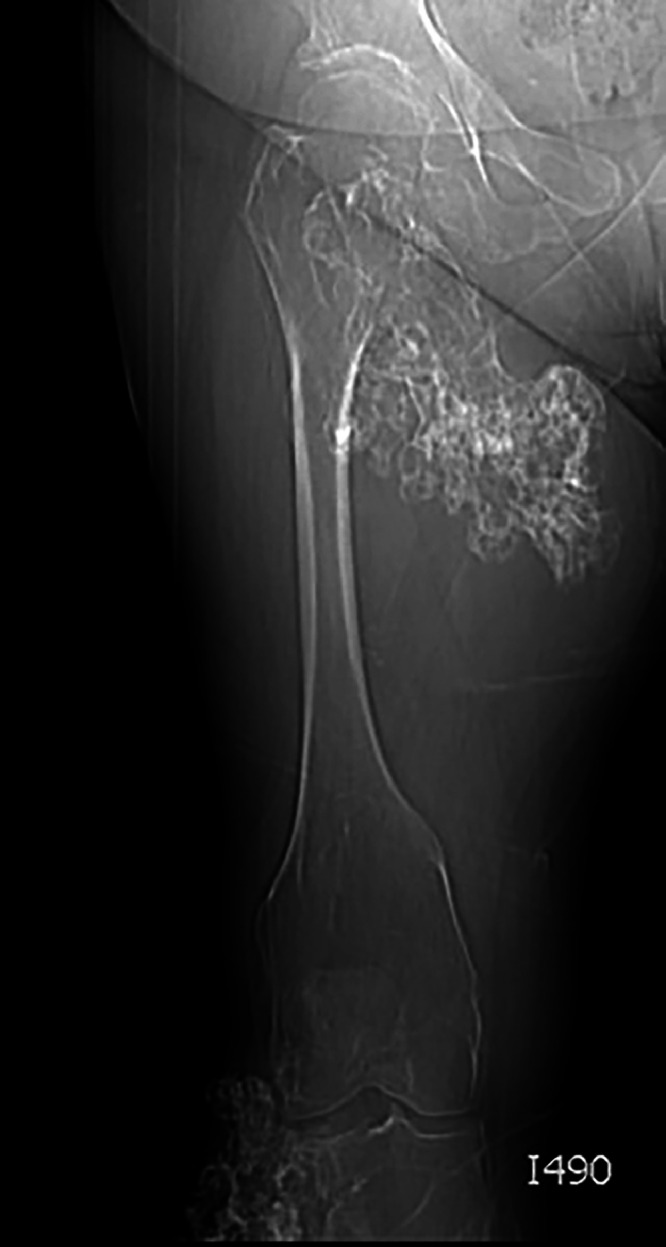
Right leg plain film, anteroposterior projection. It showed an exostosis originating from the femoral neck and involving the metaphysis. The lesion was in continuity with the medullary cavity. Margins were irregular. There were no signs of fracture. Another exophytic exostosis originated from the lateral side of the proximal tibial epiphysis.

The leg X-ray revealed the presence of a prominent exostosis away from the femoral neck. The origin was localized in the metaphysis reaching the epiphysis of the proximal femur. The lesion presented irregular margins and a cauliflower morphology. The surrounding soft tissues appeared edematous. The homolateral knee included in the study showed another prominent exostosis away from the proximal epiphysis of the tibia, on the lateral side (Fig. 3).

**Fig. 4:**
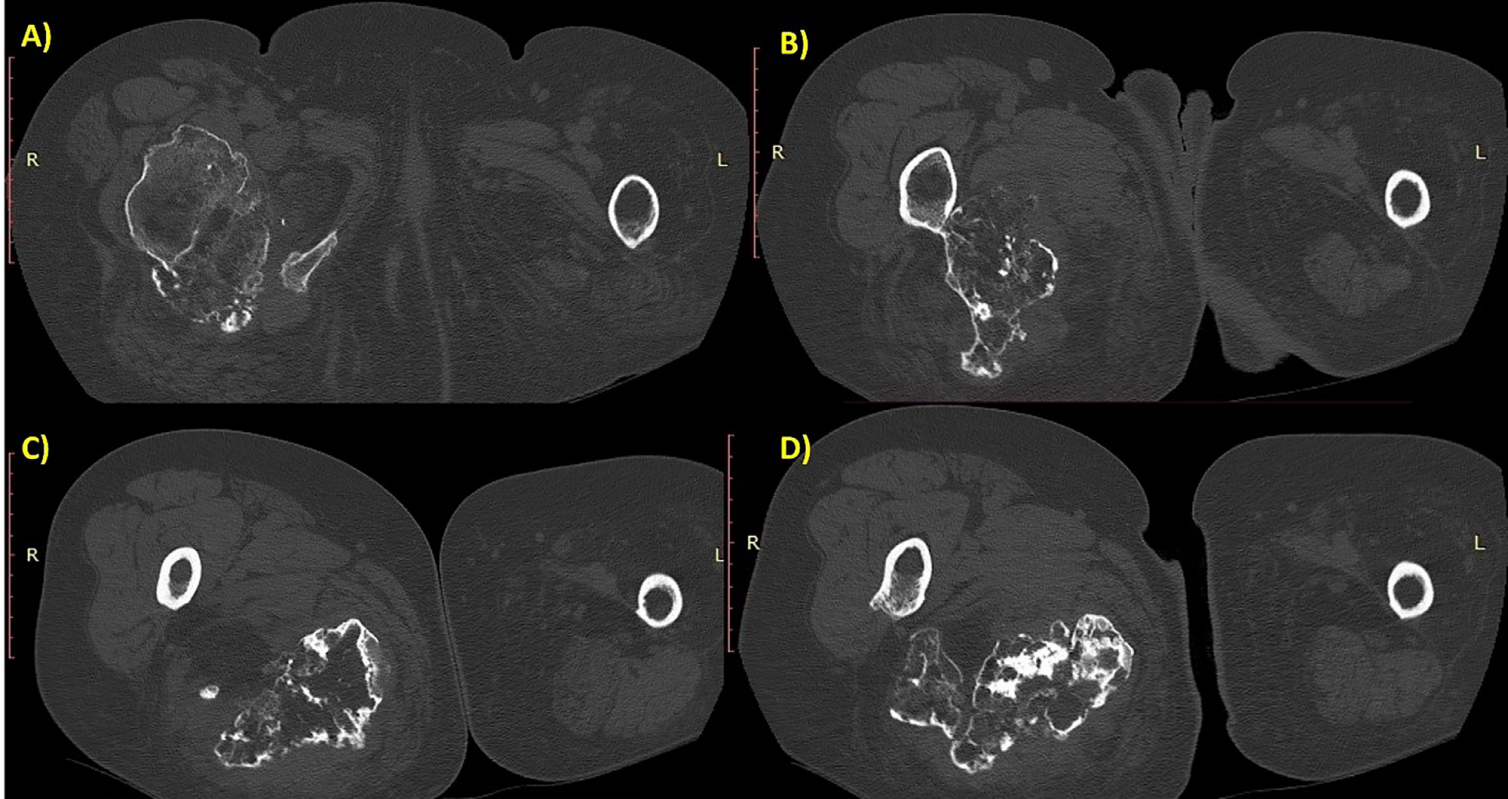
Sequential axial scan with bone window showed a giant exostosis arising from the posterior side of the femoral neck, involving the lesser trochanter, and extending into the gluteal muscles, especially the quadratus femoris.

**Fig. 5:**
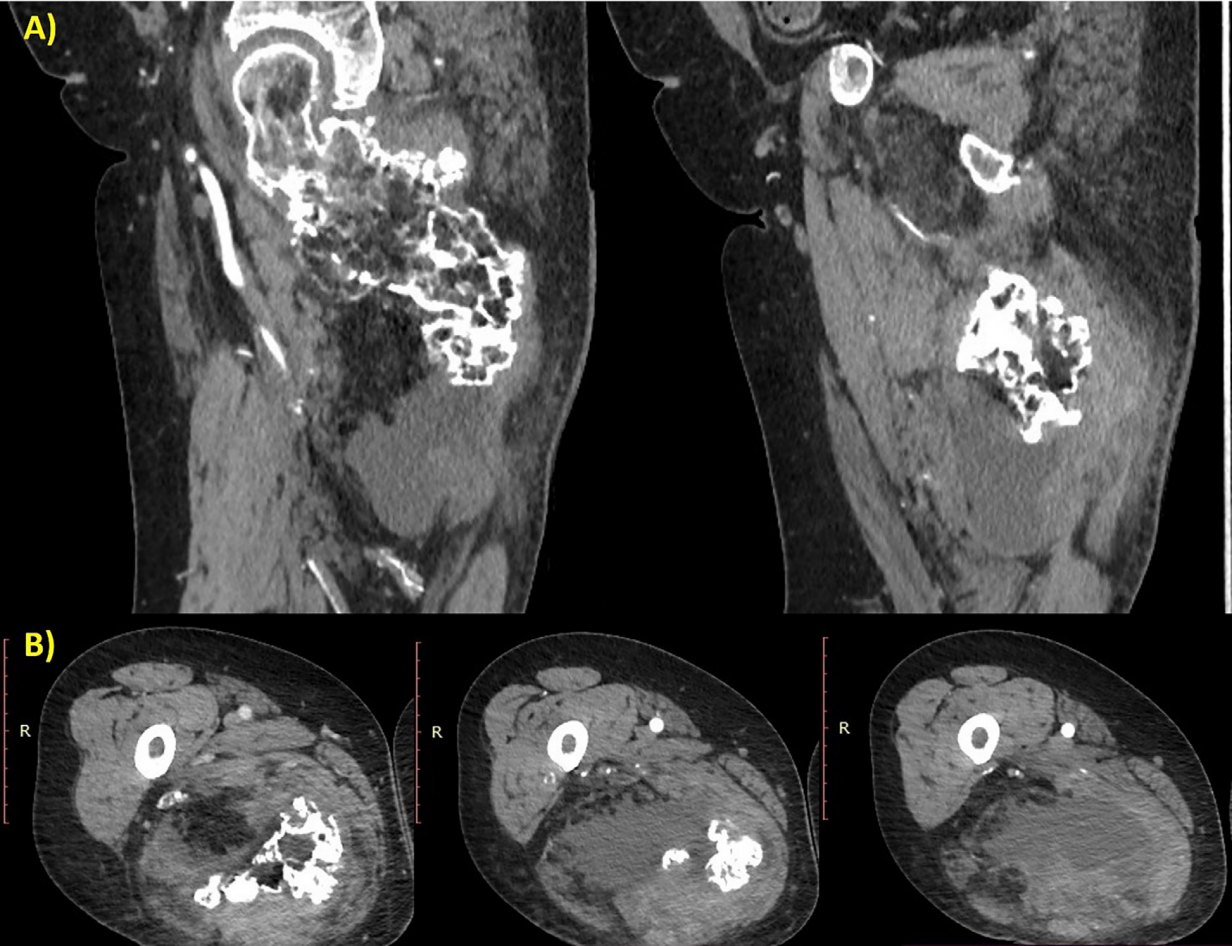
(A, B) Sequential CT scan performed after contrast agent administration in sagittal (A) and axial plane (B). In the lower part, the soft tissues were involved and showed heterogeneous density with fluid components and slight contrast enhancement. There was marked thickening of the muscle bundles of the quadratus and biceps femoris.

**Fig. 6:**
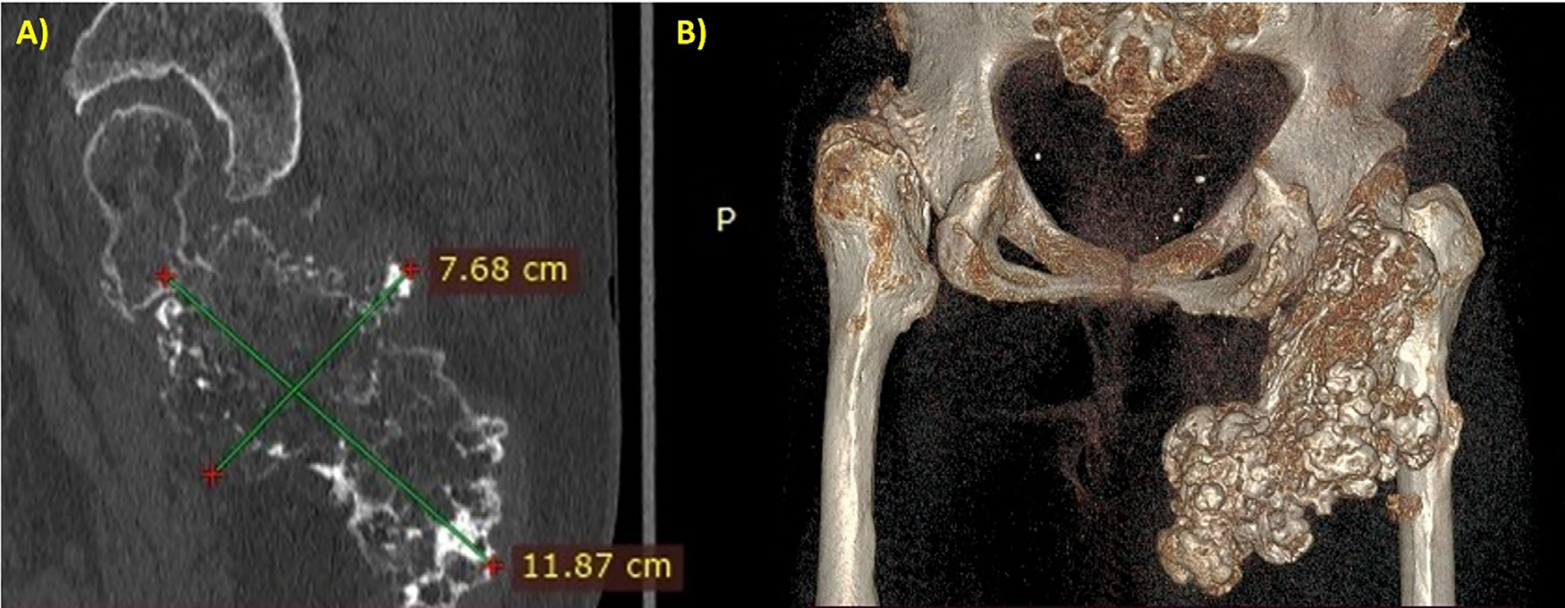
(A, B) The exostosis measured in a sagittal plane of the CT scan (A) and reconstructed in a 3D image (B).

Due to the radiological features that showed on ultrasound a mixed echogenic mass containing liquid material and on X-ray a voluminous osteochondrosis, which was clinically correlated with worsening pain, it was deemed appropriate to proceed with a CT scan with contrast medium. The CT scan performed before and after contrast administration showed a growth bone mass extended from the proximal femur, measuring about 11 × 8 cm in diameter. In addition to the voluminous calcific component, the mass also included sub-solid component, the latter with fluid and slight contrast enhancement. The surrounding soft tissues appear edematous and compressed (Fig. 4, Fig. 5, Fig. 6).

**Fig. 7:**
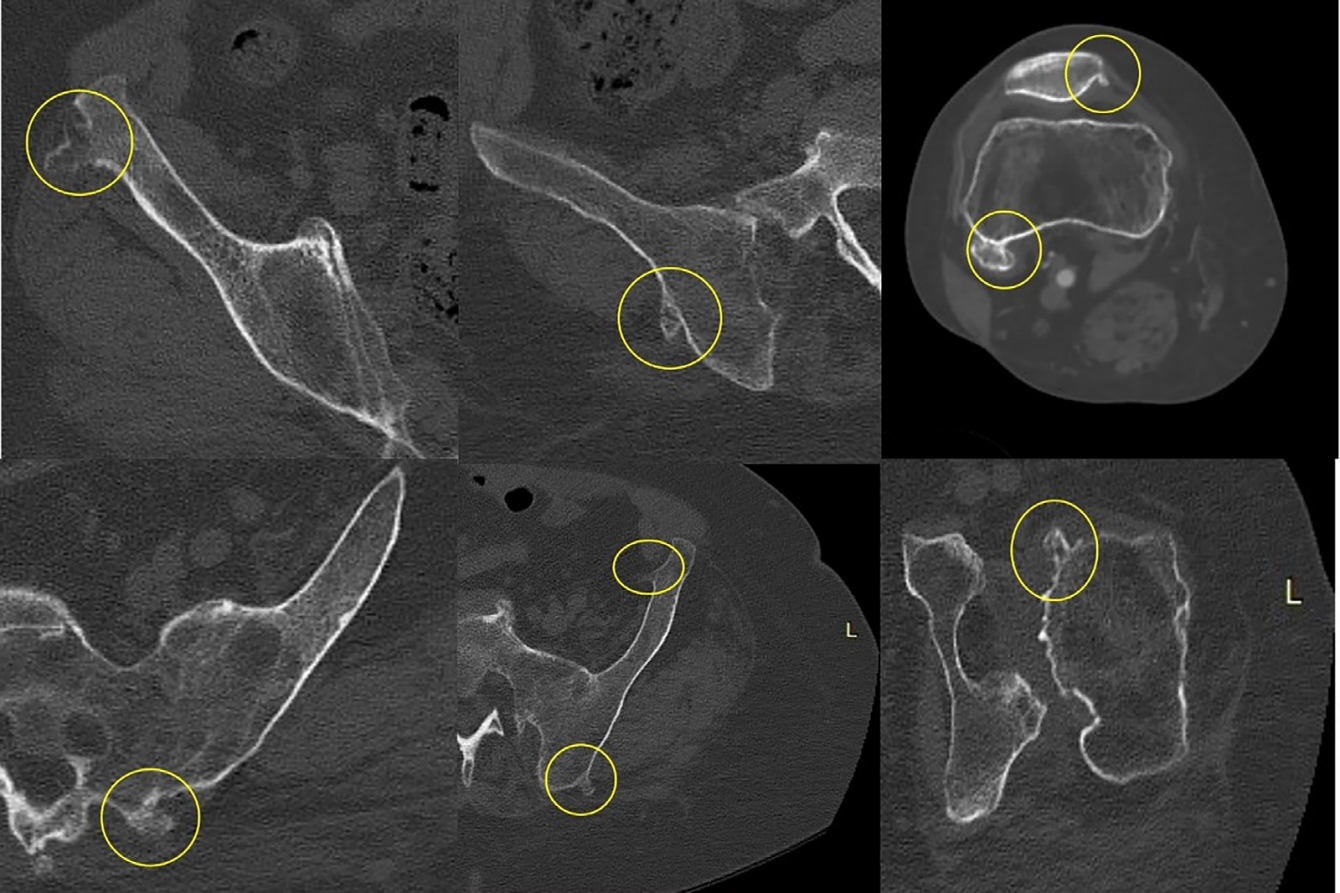
Multiple axial CT scan showing multiple sessile and pedunculated osteochondromas of the pelvis and knee. The bones involved in image order are iliac wing and body on the right, patella and femur on the right, sacral wing on the left, iliac wing and body on the left, femoral head on the left.

**Fig. 8:**
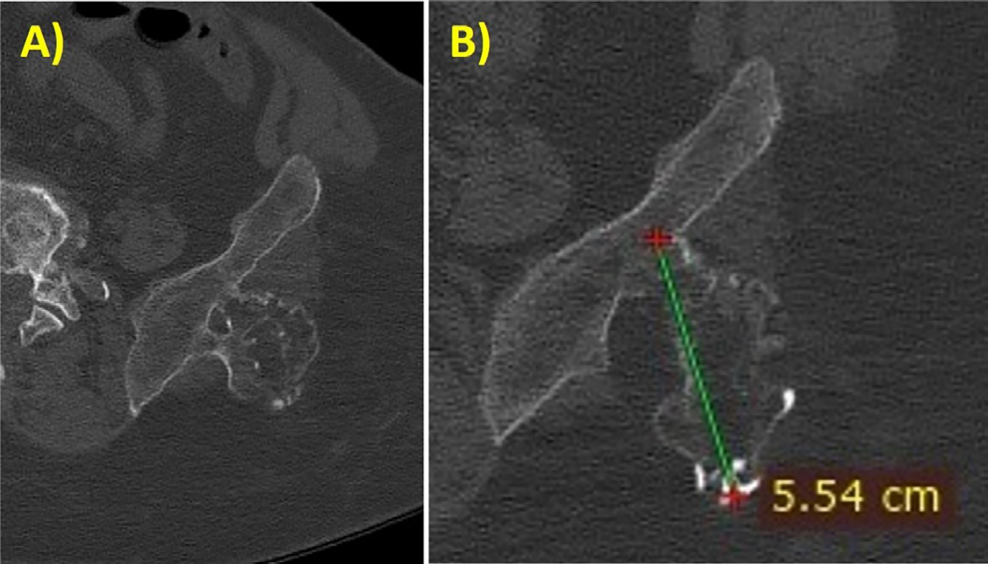
(A, B) CT axial scan showing another bulky growth-bone lesion (maximum diameter of 5 cm) originating from the left ileum.

At the CT scan, other multiple exostoses were visualized, especially at the right ilium, according to the HME (Fig. 7, Fig. 8).

The tomodensitometric features of an inhomogeneous solid component adjacent to the osteochondrosis raised the suspicion of malignancy, especially in a patient with HME that might be associated to complications of malignant degeneration. However, the presence of benign pathology, such as hematoma, could not be excluded from the radiological features reported. In the suspicion of chondrosarcoma, the imaging method that allows greater diagnostic accuracy is MRI. However, this kind of examination could not be carried out due to the lack of compliance of the patient, who suffers from claustrophobic fits.

The patient was started on analgesic medication to alleviate pain associated with the bony mass. Given the suspicious malignant nature of the lesion, the patient was referred to an orthopedic surgeon specializing in musculoskeletal oncology. The biopsy confirmed the suspected radiological findings of chondrosarcoma.

## Discussion

Chondrosarcoma is a malignant tumor derived from cartilage-forming cells and is the most common malignancy associated with HME [6,8].

The diagnosis of malignant transformation in HME requires careful clinical evaluation and imaging studies to assess the extent of bony involvement and soft tissue invasion. In the case reported, the patient's clinical presentation of right thigh pain and enlargement, combined with a known history of HME, prompted further investigation through imaging modalities (ultrasound, X-ray, and CT scan) which have raised suspicion of a chondrosarcoma malignant transformation, subsequently confirmed histologically.

The US examination usually provides additional details regarding the soft tissue involvement and vascularity surrounding the lesions. Due to the volume of the lesion, it was not easy to recognize the origin of the lesion and to evaluate the cartilage cap which is usually shown as a hypoechoic area superficially limited by muscle and fat and deeply bounded by bone. It was not possible to delineate the lesion's margins and to separate the different types of components. The ultrasound examination, with its limitation in assessing bone structures but raising concern for possible malignancy, required further investigation.

The plain radiograph showed the main features of osteochondroma with the common origin in the meta-epiphyseal region of the proximal femur protruding out with a cauliflower morphology.

The CT scan is useful to assess the bone matrix, see the medullary continuity, and estimate the soft tissue involvement. In the case reported, CT scans performed before and after contrast administration provided further characterization of the mass, revealing features highly suggestive of chondrosarcoma. The bone window showed the precise localization and extension of the lesion, as well as the cortical remodeling and the periosteal reaction. The soft tissue window showed another component of the mass, in particular, the fluid and subsolid parts with slight contrast enhancement. The surrounding soft tissues appeared compressed by the mass and edematous. The exostosis indents and distorts the gluteal muscles, in particular the quadratus femoris.

The CT findings supported voluminous osteochondrosis in the context of HME, associated with involvement of the soft tissues compatible with hematoma but which, related to the patient's anamnestic history and symptoms, increased the suspicion of malignant degeneration of the osteochondroma. Considering that it was not possible to carry out an MRI and obtain greater diagnostic accuracy, the contrast-enhanced CT nevertheless made it possible to detect the presence of solid tissue, with enhancement, of non-univocal significance and of such a suspicious nature as to require further investigation diagnostic. Malignant transformation to chondrosarcoma was subsequently confirmed by histological examination.

The histological evaluation grades the chondrosarcoma from 1 to 3, considering the nuclear size, staining pattern, mitotic activity, and cellularity degree. Furthermore, the non-mineralized tissue would exhibit a high-water content and change histologically from a more myxoid stroma to mature hyaline cartilage. Chondrosarcoma margins are distinguished by the invasion of trabecular bone by chondroid tissue. Extraosseous extension begins with invasion of the endosteal surface, which is the initial stage of high-grade chondrosarcoma [9].

The WHO's Fifth Edition classification of soft tissue and bone tumors specifies the following diagnostic criteria for chondrosarcoma: 1) cartilaginous cap >2 cm; 2) underlying stalk with medullary and cortical continuation with underlying bone; 3) growth-like architecture in youngsters or significant calcification with age [2,10]. In the case reported it was not possible to evaluate the criteria listed above as they require an MRI study. In particular, measuring the cartilage cup on MRI is a consistent technique for predicting osteochondromas' malignant development. The measurement should be taken from the exostosis pedicle's bony interface to the cartilaginous cap's thickest part's edge. Adults with more than 1-2 cm thicknesses should be suspected of malignant degeneration [7]. As in the reported case, additional radiographic indicators of malignant transformation include soft tissue mass around the exostosis, evaluable on CT. As a result, when a radiological image suggests malignant degeneration, additional research is necessary to get a histology-based definite diagnosis.

The risk of malignant transformation is higher in patients with HME, necessitating vigilant monitoring and timely intervention. Treatment of malignant transformation in HME typically involves a multidisciplinary approach, including surgical resection of the tumor with wide margins, adjuvant radiation therapy, and chemotherapy in certain cases. In the case reported the biopsy performed revealed a differentiated chondrosarcoma, which required a surgical excision: the exostosis at the bone base and the cartilage cap were removed. The prognosis depends on various factors, including the histologic grade of the tumor, the extent of the disease, and the adequacy of surgical resection [6,11].

## Conclusion

This case highlights the importance of recognizing the potential for malignant transformation in patients with HME. Early detection through imaging plays a crucial role in guiding treatment decisions and optimizing patient outcomes.

## Authors contribution

The authors confirm contribution to the paper as follows: study conception and design: FM Author, MM Author, RC Author; data collection: FM Author, MB Author, RC Author; analysis and interpretation of results: FM Author, GF Author, RG Author, GG Author; draft manuscript preparation: FM Author, MM Author, MB Author. All authors reviewed the results and approved the final version of the manuscript.

## Patient consent

Complete written informed consent was obtained from the patient for the publication of this study and accompanying images.
